# Relationships among emotional intelligence, conscientiousness, and resilience in nurses at Hamadan teaching hospitals, Iran: a cross-sectional study

**DOI:** 10.1186/s12912-026-05131-8

**Published:** 2026-07-30

**Authors:** Meysam Safi-Keykaleh, Parisa Solgi, Hamid Safarpour, Maryam Maddineshat, Sajad Zivari-Nemat, Mohammad Torabi

**Affiliations:** 1 Nahavand School of Medical Sciences, Hamadan University of Medical Sciences, Hamadan, Iran; 2 Department of Health in Disasters and Emergencies, Ilam University of Medical Sciences, Ilam, Iran; 3 Malayer School of Medical Sciences, Hamadan University of Medical Sciences, Hamadan, Iran; 4 Master of Medical Surgical Nursing, Hamadan University of Medical Sciences, Hamadan, Iran

**Keywords:** Emotional intelligence, Conscientiousness, Resilience, Nurses, Iran

## Abstract

**Background:**

Nurses working in high-stress environments depend substantially on resilience to maintain mental health and professional performance. Emotional intelligence (EI) and conscientiousness are known to be associated with resilience; however, their interrelationships in Iranian hospital nurses have not been thoroughly investigated. Therefore, this study aimed to investigate the relationships among EI, conscientiousness, and resilience in nurses at teaching hospitals in Hamadan, Iran.

**Methods:**

This cross-sectional study was conducted in 2025 on 390 nurses from nine teaching hospitals in Hamadan, Iran, using proportional stratified random sampling. Data were collected using the Wong and Law Emotional Intelligence Scale (WLEIS), the conscientiousness subscale of the NEO Five-Factor Inventory (NEO-FFI), and the 10-item Connor-Davidson Resilience Scale (CD-RISC-10). Descriptive statistics, Pearson correlation analysis, and hierarchical multiple regression analysis were conducted using SPSS version 26.

**Results:**

There was a moderately strong positive association between EI and resilience (*r* = 0.56, *p* < 0.001) and between conscientiousness and resilience (*r* = 0.49, *p* < 0.001). The final regression model, including demographic variables, EI, and conscientiousness, explained 47% of the variance in resilience (R² = 0.47, F(7, 382) = 48.30, *p* < 0.001), with EI showing the strongest association with resilience.

**Conclusions:**

EI and conscientiousness are significantly associated with resilience in this sample of nurses. Assessing these psychological resources and evaluating targeted training programs may be useful for supporting resilience in high-stress clinical settings. However, longitudinal and interventional studies are needed to confirm these findings.

## Background

Resilience, defined as the ability to effectively adapt to and recover from stressful situations, is a key determinant of mental health and professional performance among nurses in high-pressure environments [1, 2]. The World Health Organization has warned that inadequate support for nurses’ psychological resilience may exacerbate a projected global shortage of 18 million nurses by 2030, primarily because of burnout and high turnover rates [3]. Longitudinal and cross-sectional studies conducted between 2020 and 2025 have consistently shown that highly resilient nurses experience significantly lower burnout (up to 45% reduction), higher job satisfaction, lower turnover intention (OR = 0.41), and better quality of patient care [4–8]. This issue is particularly important in low- and middle-income countries, such as Iran, where the nurse-to-population ratio is approximately 1.6 per 1,000 population and more than 68% of nurses are female [9, 10].

Iranian nurses have reported lower levels of resilience compared to their counterparts in many other countries. This difference may be attributed to challenges such as long working hours, persistent workload pressures following the COVID-19 pandemic, and resource scarcity [11–13]. This disparity underscores the need to identify modifiable factors associated with resilience.

Among the established factors associated with resilience, EI and conscientiousness have emerged as particularly important. EI, conceptualized as the ability to perceive, understand, regulate, and utilize emotions in oneself and others, is associated with higher resilience through adaptive emotion regulation, better stress management, and stronger social support networks [14, 15]. Similarly, conscientiousness, a core dimension of the Big Five personality model, reflects traits such as self-discipline, organization, responsibility, and goal-directed behavior. These characteristics are associated with proactive coping strategies, persistence in the face of adversity, and effective problem-solving, which may contribute to higher levels of resilience [15, 16].

Examining EI and conscientiousness simultaneously is theoretically valuable because these constructs represent complementary pathways to resilience. EI reflects a relatively malleable, emotion-focused capacity, whereas conscientiousness represents a more stable, behavior-oriented personality trait. Investigating both constructs within a single model may provide a more comprehensive understanding of how emotional and personality factors are jointly associated with resilience. Although there is strong evidence supporting the independent associations of EI and conscientiousness with resilience, few studies have examined all three constructs simultaneously in a single model. Previous studies in Jordan, Saudi Arabia, South Africa, and China have primarily examined pairwise relationships (e.g., EI-resilience or conscientiousness-burnout) and have not simultaneously investigated the combined associations of EI and conscientiousness on resilience [7, 8, 17, 18]. To date, no study in Iran has comprehensively examined the relationships among EI, conscientiousness, and resilience in hospital nurses. Therefore, the present study aimed to investigate the relationships among EI, conscientiousness, and resilience in nurses working in teaching hospitals in Hamadan, Iran, in 2025.

## Methods

### Study design and setting

This cross-sectional study was conducted in 2025 at nine teaching hospitals affiliated with Hamadan University of Medical Sciences, Iran (Be’sat, Farshchian, Ekbatan, Shahid Beheshti, Fatemiyeh, Imam Hossein, Mehr, Ayatollah Alimoradin, and Shahid Ghodosi Hospitals).

### Population, sample size, and sampling method

The target population consisted of approximately 2,000 clinical nurses employed in the nine teaching hospitals. Cochran’s formula for finite populations was used to calculate the sample size:

$$\begin{aligned}\mathrm{n}&={\mathrm{[N}\times \mathrm{z}^2 \times \mathrm{p}\,(1-\mathrm{p})]}\cr&\quad/[\mathrm{d}^2\,(\mathrm{N}-1)+\mathrm{z}^2 \times \mathrm{p}\,(1-\mathrm{p})]\end{aligned}$$

Assuming *N* = 2000, z = 1.96 (95% confidence level), *p* = 0.5, d = 0.05, the minimum required sample size was 384. To allow for a 10% dropout rate, 423 questionnaires were distributed. Ultimately, 390 completed questionnaires were returned and analyzed (response rate = 92%).

Proportional stratified random sampling was employed, with strata defined according to hospital affiliation. The number of participants allocated to each hospital was proportional to the hospital’s total number of clinical nurses. Within each stratum, participants were randomly selected using current staff lists obtained from the hospital nursing administration as the sampling frame. A total of 33 questionnaires were excluded due to non-response or incomplete data (more than 10% missing items). A complete-case analysis was performed. Prior to the main analysis, Little’s Missing Completely at Random (MCAR) test indicated that the missing data were completely at random (*p* > 0.05).

### Inclusion and exclusion criteria

Inclusion criteria were: (a) clinical nurses working in inpatient wards, and (b) at least one year of clinical nursing experience.

Exclusion criteria were: (a) working in non-clinical departments, or (b) incomplete questionnaires (> 10% missing items).

### Data collection instruments

A demographic questionnaire was used to collect data on age, gender, marital status, level of education, years of experience, and ward type.

The WLEIS (Wong and Law, 2002) comprises 16 items organized into four dimensions (self-emotion appraisal, others’ emotion appraisal, use of emotion, and regulation of emotion). Responses are rated on a 7-point Likert scale (1 = strongly disagree to 7 = strongly agree). This instrument was not developed specifically for the current study. The Persian version has previously been validated among Iranian nurses, with Cronbach’s alpha coefficients ranging from 0.89 to 0.91 [19, 20]. In the present study, Cronbach’s alpha was 0.91.

The conscientiousness subscale (Costa and McCrae, 1992) contains 12 items rated on a 5-point Likert scale (0 = strongly disagree, 4 = strongly agree) with a maximum total score of 48. This subscale was not developed for the current study. The validated Persian version has previously been used in Iranian studies. Cronbach’s alpha coefficients in Iranian studies have been found to be 0.83 to 0.87 [21, 22]. In the present study, Cronbach’s alpha was 0.88.

Connor-Davidson Resilience Scale-10 (CD-RISC-10) (Campbell-Sills and Stein, 2007) consists of 10 items rated on a 5-point Likert scale (0 = strongly disagree to 4 = strongly agree), with a total score range of 0–40. This scale was not developed for the current study. The validated Persian version has previously been used in Iranian nursing research. Cronbach’s alpha coefficients reported in Iranian nursing studies have ranged from 0.88 to 0.93 [23, 24]. In the present study, Cronbach’s alpha was 0.90.

### Validity and reliability

The Persian versions of the WLEIS, NEO-FFI conscientiousness subscale, and CD-RISC-10 have been previously validated in Iranian populations. In the current study, the internal consistency of all instruments was acceptable (Cronbach’s alpha = 0.91 for WLEIS, 0.88 for the conscientiousness subscale, and 0.90 for CD-RISC-10).

### Data collection procedure

The researcher attended the participating hospitals during morning and evening shifts after obtaining official permission. After explaining the study objectives and ensuring confidentiality, informed written consent was obtained from all participants. Participation was completely voluntary and anonymous.

### Statistical analysis

Data were analyzed using SPSS version 26. Descriptive statistics were computed for all variables. The Kolmogorov-Smirnov test indicated that the data were normally distributed. Pearson’s correlation and hierarchical multiple regression analyses were conducted to examine the associations among study variables and identify factors associated with resilience. All regression assumptions, including linearity, homoscedasticity, normality of residuals, independence of errors, and multicollinearity, were checked and met. Categorical variables included in the regression model (gender, marital status, and education level) were dummy-coded before analysis. Due to the small number of clusters (nine hospitals) and low intra-class correlation, multilevel modeling was not performed, and single-level hierarchical regression was used. Statistical significance was set at *p* < 0.05. Variance inflation factors (VIFs) were examined, and all VIF values were < 2.0.

## Results

### Participants’ demographic characteristics

A total of 390 nurses participated in the study (Table 1). The mean age was 34.2 years (SD = 7.1, range: 23–55), and the mean clinical experience was 9.4 years (SD = 5.6, range: 1–30). The Kolmogorov-Smirnov test confirmed that all study variables were normally distributed (*p* > 0.05).

**Table 1:** Demographic characteristics of the participants (*N* = 390) on study of the relationships among EI, conscientiousness, and resilience among nurses in Hamadan teaching hospitals, Iran: a cross-sectional study

Characteristic	Category	*n*	%	Mean (SD)
Gender	Female	265	68.0	-
	Male	125	32.0	-
Marital status	Married	268	68.7	-
	Single	122	31.3	-
Education level	Bachelor’s degree	312	80.0	-
	Master’s degree or higher	78	20.0	-
Years of experience	-	-	-	9.4 (5.6)
Age (years)	-	-	-	34.2 (7.1)
Ward type	Medical-surgical	142	36.4	-
	Intensive care	98	25.1	-
	Emergency	76	19.5	-
	Others	74	18.9	-

Footnote: Age and years of clinical experience showed weak positive correlations with resilience (age: *r* = 0.18, *p* < 0.001; years of experience: *r* = 0.22, *p* < 0.001)

No significant differences were found in the main study variables according to gender, marital status, or ward type (*p* > 0.05). However, both age and years of experience were weakly and positively correlated with resilience (age: *r* = 0.18, *p* < 0.001; years of experience: *r* = 0.22, *p* < 0.001).

### Descriptive statistics for EI, conscientiousness, and resilience

The mean EI score on the WLEIS was 82.6 (SD = 13.4, range 48–110). The subscale means were as follows: self-emotion appraisal (M = 20.8, SD = 4.2), others’ emotion appraisal (M = 21.4, SD = 4.1), use of emotion (M = 20.5, SD = 3.9), and emotion regulation (M = 19.9, SD = 4.0).

The mean conscientiousness score was 34.8 (SD = 7.2, range 18–47). The mean resilience score on the CD-RISC-10 was 26.3 (SD = 6.9, range 10–38).

### Correlations among study variables

Pearson correlation coefficients were used to examine the relationships between EI, conscientiousness, and resilience. EI was positively correlated with both conscientiousness (*r* = 0.47, *p* < 0.001) and resilience (*r* = 0.56, *p* < 0.001). There was also a positive correlation between conscientiousness and resilience (*r* = 0.49, *p* < 0.001). The observed correlations were all moderate (Table 2).

**Table 2:** Pearson correlation coefficients among EI, conscientiousness, and resilience (*N* = 390)

Variable	EI	Conscientiousness	Resilience
EI	1.00	0.47^**^	0.56^**^
Conscientiousness	0.47**	1.00	0.49**
Resilience	0.56**	0.49**	1.00

Footnote: ** *p* < 0.001 for all reported correlations

### Hierarchical multiple regression analysis

Hierarchical multiple regression analysis was performed to examine the associations of the study variables with resilience. As shown in Table 3, in Step 1, the demographic variables explained 6% of the variance in resilience (R² = 0.06), with the overall model being statistically significant (F (5, 384) = 4.92, *p* < 0.001), and years of experience was the only demographic variable significantly associated with resilience (β = 0.19, *p* = 0.003).

**Table 3:** Summary of hierarchical multiple regression analysis of factors associated with resilience (*N* = 390)

Step and Predictor	β	SE	t	*p*-value	*R*²	ΔR²	Overall F	F-change
**Step 1: Demographics**					0.06	0.06	F(5,384) = 4.92^***^	
Age	0.12	0.08	1.82	0.070				
Gender (male = ref.)	-0.05	0.92	-0.78	0.436				
Marital status (single = ref.)	0.08	0.74	1.24	0.216				
Education (Bachelor’s = ref.)	0.10	0.85	1.56	0.120				
Years of experience	0.19	0.09	2.98	0.003^**^				
**Step 2. EI**					0.35	0.29	F(6,383) = 34.6^***^	168.4^***^
EI	0.54	0.03	12.98	< 0.001^***^				
**Step 3. Conscientiousness**					0.47	0.12	F(7,382) = 48.30***	82.6^***^
Conscientiousness	0.38	0.04	9.09	< 0.001^***^				

Footnote: β = standardized regression coefficient; SE = standard error; R² = coefficient of determination; ΔR² = change in the coefficient of determination. The F values reported include both the overall model F statistics and the F-change statistics for each step of the hierarchical regression model. Categorical variables included in the regression model (gender, marital status, and education level) were dummy-coded before analysis. Reference categories were: Gender (Male = 0), Marital status (Single = 0), and Education level (Bachelor’s degree = 0). All variance inflation factor (VIF) values were < 2.0, indicating no evidence of multicollinearity. **p < 0.01; ***p < 0.001

β = standardized coefficient; SE = standard error

In Step 2, adding EI to the model significantly increased the explained variance by 29% (ΔR² = 0.29, F-change = 168.4, *p* < 0.001; β = 0.54, *p* < 0.001). In Step 3, the addition of conscientiousness further increased the explained variance by 12% (ΔR² = 0.12, F- change = 82.6, *p* < 0.001; β = 0.38, *p* < 0.001). The final model accounted for 47% of the variance in resilience (R² = 0.47, F (7, 382) = 48.3, *p* < 0.001). No evidence of multicollinearity was detected (all VIF values were < 2.0) (Table 3).

## Discussion

This study showed that resilience had a moderate-to-strong positive association with both EI (*r* = 0.56, *p* < 0.001) and conscientiousness (*r* = 0.49, *p* < 0.001). Hierarchical multiple regression analysis revealed that, after controlling for demographic variables, EI and conscientiousness together accounted for 47% of the variance in resilience (R² = 0.47). EI showed the strongest association with resilience, explaining an additional 29% of the variance, while conscientiousness contributed an additional 12%. These results indicate significant positive associations between EI, conscientiousness, and resilience. The mean resilience score in this sample was 26.3 (SD = 6.9) out of 40, which is consistent with previous Iranian studies reporting moderate resilience levels among nurses [13, 25]. The positive association between EI and resilience is consistent with systematic reviews and meta-analyses that have shown that EI training has been reported to improve resilience and reduce stress levels among nurses in intervention studies [14, 26]. Similar correlations between EI and resilience have been reported in cross-sectional studies [27, 28].

Regarding conscientiousness, the findings are consistent with the Big Five personality model, in which this relatively stable trait is positively associated with resilience through characteristics such as diligence, organization, and proactive problem-solving [29, 30]. These findings are consistent with previous studies showing that conscientiousness, along with EI, is associated with better coping behaviors and resilience among nurses [31]. Similar associations between EI and conscientiousness have also been reported among Iranian nurses [32]. However, some differences were observed when compared with international studies. Moreover, the mean resilience score in our sample (26.3 out of 40) was lower than that reported in several studies from other countries [17, 33]. These differences may be attributed to contextual factors such as longer working hours, resource limitations following the COVID-19 pandemic, and the relatively low nurse-to-population ratio in Iran [13, 34]. In contrast, nurses in high-resource settings may have greater access to psychological support and resilience training programs. Similar patterns of reduced resilience and increased anxiety were also observed among Iranian nurses working in COVID-19 wards, highlighting the role of organizational support during crises [35].

The current study found weak positive associations between resilience and both age (*r* = 0.18, *p* < 0.001) and years of experience (*r* = 0.22, *p* < 0.001), which differs from some previous findings suggesting that younger nurses tend to have lower resilience due to limited experience [23]. These discrepancies may be attributed to variations in the measurement instrument (e.g., CD-RISC-10 versus longer scales) or sample characteristics.

Additionally, no significant differences were observed in EI, conscientiousness, or resilience according to gender, marital status, or ward type. However, this finding should be interpreted with caution because 68% of the sample was female. This result aligns with several Iranian studies reporting no gender differences in EI among clinical nurses [36]. Furthermore, the mean conscientiousness score in this sample (34.8 out of 48) was relatively high, which is consistent with previous personality research describing conscientiousness as a common trait among nurses.

The present findings have practical implications for nursing managers and hospital administrators in similar settings. Given the observed associations, routine screening for EI and resilience levels may help identify nurses who might benefit from additional support. Furthermore, implementing resilience-building programs and EI training as part of professional development could be useful strategies for supporting nurses’ ability to cope with high-stress clinical environments. These recommendations are based on the observed associations and should be viewed as potential strategies that require further evaluation through longitudinal and interventional studies.

### Limitations

This study has several limitations. First, the cross-sectional design precludes causal inferences. Second, the sample was limited to nurses from teaching hospitals in Hamadan, which may limit the generalizability of the findings. Third, although the response rate was high (92%), non-response bias cannot be entirely ruled out. Fourth, the use of self-report measures may have introduced common method bias; however, anonymous questionnaires were used to minimize this possibility. Despite these limitations, the findings provide valuable insights into the relationships between EI, conscientiousness, and resilience among Iranian nurses, which have been insufficiently explored.

## Conclusion

The present study found significant positive associations between emotional intelligence (EI), conscientiousness, and resilience among nurses in teaching hospitals in Hamadan, Iran. EI demonstrated the strongest association with resilience. The moderate level of resilience observed in this sample underscores the potential importance of psychological factors in high-stress clinical environments.

Given these findings, nursing managers may consider assessing emotional intelligence and resilience as part of broader workforce support strategies and exploring targeted educational initiatives aimed at strengthening these attributes. However, these implications should be interpreted with caution, as the cross-sectional design precludes causal inference. This study contributes to the limited evidence on the simultaneous associations of EI and conscientiousness with resilience among Iranian nurses. Future longitudinal and interventional studies are needed to determine whether interventions targeting these psychological characteristics can effectively enhance resilience and to identify additional factors that may influence these relationships.

## Data Availability

The datasets generated and analyzed during the current study are available from the corresponding author on reasonable request due to privacy and ethical restrictions.

## Abbreviations

WLEIS: Wong and Law EI Scale
NEO-FFI: NEO Five-Factor Inventory
CD-RISC-10: 10-item Connor-Davidson Resilience Scale
